# Temporal quality degradation in AI models

**DOI:** 10.1038/s41598-022-15245-z

**Published:** 2022-07-08

**Authors:** Daniel Vela, Andrew Sharp, Richard Zhang, Trang Nguyen, An Hoang, Oleg S. Pianykh

**Affiliations:** 1 Monterrey Institute of Technology and Higher Education, Monterrey, Mexico; 2 Massachusetts Institute of Technology, Cambridge, USA; 3 Whitehead Institute for Biomedical Research, Cambridge, USA; 4 Harvard University Medical School, Boston, USA

**Keywords:** Health care, Mathematics and computing, Applied mathematics, Scientific data

## Abstract

As AI models continue to advance into many real-life applications, their ability to maintain reliable quality over time becomes increasingly important. The principal challenge in this task stems from the very nature of current machine learning models, dependent on the data as it was at the time of training. In this study, we present the first analysis of AI “aging”: the complex, multifaceted phenomenon of AI model quality degradation as more time passes since the last model training cycle. Using datasets from four different industries (healthcare operations, transportation, finance, and weather) and four standard machine learning models, we identify and describe the main temporal degradation patterns. We also demonstrate the principal differences between temporal model degradation and related concepts that have been explored previously, such as data concept drift and continuous learning. Finally, we indicate potential causes of temporal degradation, and suggest approaches to detecting aging and reducing its impact.

## Introduction

Artificial Intelligence (AI), and machine learning (ML) models in particular, are becoming increasingly present in many real-life applications, from finance^1,2^ and manufacturing^3^ to agriculture^4^ and healthcare^5,6^. As these practical uses multiply, the applied aspects of sustainable AI model quality become increasingly important, calling for more efforts to make AI implementations dependable and robust.

The principal challenge in maintaining AI model quality stems from the very nature of the current ML models. Trained from and driven by data, these models become inherently dependent on the data as it was *at the time of training*. We can loosely divide this model-data dependency into two principal types: *location-specific* and *time-specific*.

Model dependency on the training data *location* has been studied to a great extent, and several approaches have already been proposed to minimize its impact on model quality. For example, *federated learning* allows models to be trained, validated, and shared between different sites with varying data patterns^7^. *Transfer learning* gives additional training to the model when the model is moved to a new environment^8^. *AI bias* tests help identify and stop the propagation of data bias and “shortcuts” into ML models^9,10^. As a result, it is becoming more and more common to apply these tools to newly trained models, to make sure they can correctly scale to their diverse environments^11–13^.

Model dependency on *time*, on the contrary, has been virtually ignored in practical AI implementations: it is commonly assumed that once a model has been trained to achieve the required quality, it is ready to be deployed and used without further updates or retraining. However, data-producing environments^14–16^, often change with time, and their statistical properties change alongside them. Known as “concept drift”, this evolution of data inevitably affects the quality of the models, to the point where the model may no longer correspond to its new reality^17–19^. While much research has been done on various types and markers of temporal data drifts, there is no comprehensive study of how the models themselves can respond to these drifts.

Therefore, from the model quality perspective, *temporal model degradation* introduces a completely new challenge, which we would like to refer to as “*AI aging*”. Even though *continuous* and *online learning* have been discussed for decades as the principal means to keep AI models in sync with their environments^20^, we still lack any systematic knowledge on what exactly should trigger and control model retraining, and whether it is needed at all^21^. At the same time, our own experiences with continuously-learning models suggest that temporal model degradation can be very significant, even in environments with minimal concept drifts^22^. This was also confirmed by recent reports from different AI application fields including finance^23^, marine science^24^, and industrial predictive maintenance^25^. Only recently have some industries, such as healthcare, started recognizing the temporal aspects of AI model quality^26,27^.

Therefore, to start addressing this problem systematically, we present this study as the first analysis of the main AI temporal degradation patterns. We start this analysis by introducing a testing framework for identifying temporal model degradation. We then apply this test to 32 datasets with sufficient longitudinal components, using 4 standard AI models, to investigate how temporal model degradation can develop even under minimal drifts in the data, and under these known-to-be robust models. This enables us to identify several major temporal degradation patterns, ranging from rather anticipated to completely unexpected.

Our results demonstrate that temporal model degradation should be considered as a new, separate phenomenon, which can be triggered, but not entirely explained, by the underlying data concept drifts. We discuss the practical implications of this phenomenon, and ways to control temporal model quality in real-life applications.

## Materials and methods

### Choice of models and data

Our principal goal was to study temporal model degradation patterns present in the most standard machine learning algorithms, commonly used in ML/AI projects. Therefore, we have chosen the following 4 major model types, incorporating four principal approaches to ML model design (linear models, ensembles, boosted models, and neural networks)^28^:

1. *Penalized linear regression* RidgeRegressor model (RV).
2. *Random forest* RandomForestRegressor model (RF).
3. *Gradient boosting* XGBoost model (XG).
4. *Neural network* MLPerceptronRegressor model (NN).

We intentionally selected these model types as representing classical yet entirely different mathematical approaches to ML model design and training, and incorporating well-established optimization and regularization algorithms ensuring stability to unseen data, noise, and overfitting. By comparing aging trends across these model types, we were able to investigate similarities and differences in the ways that different models can age on the same data.

**Figure 1:**
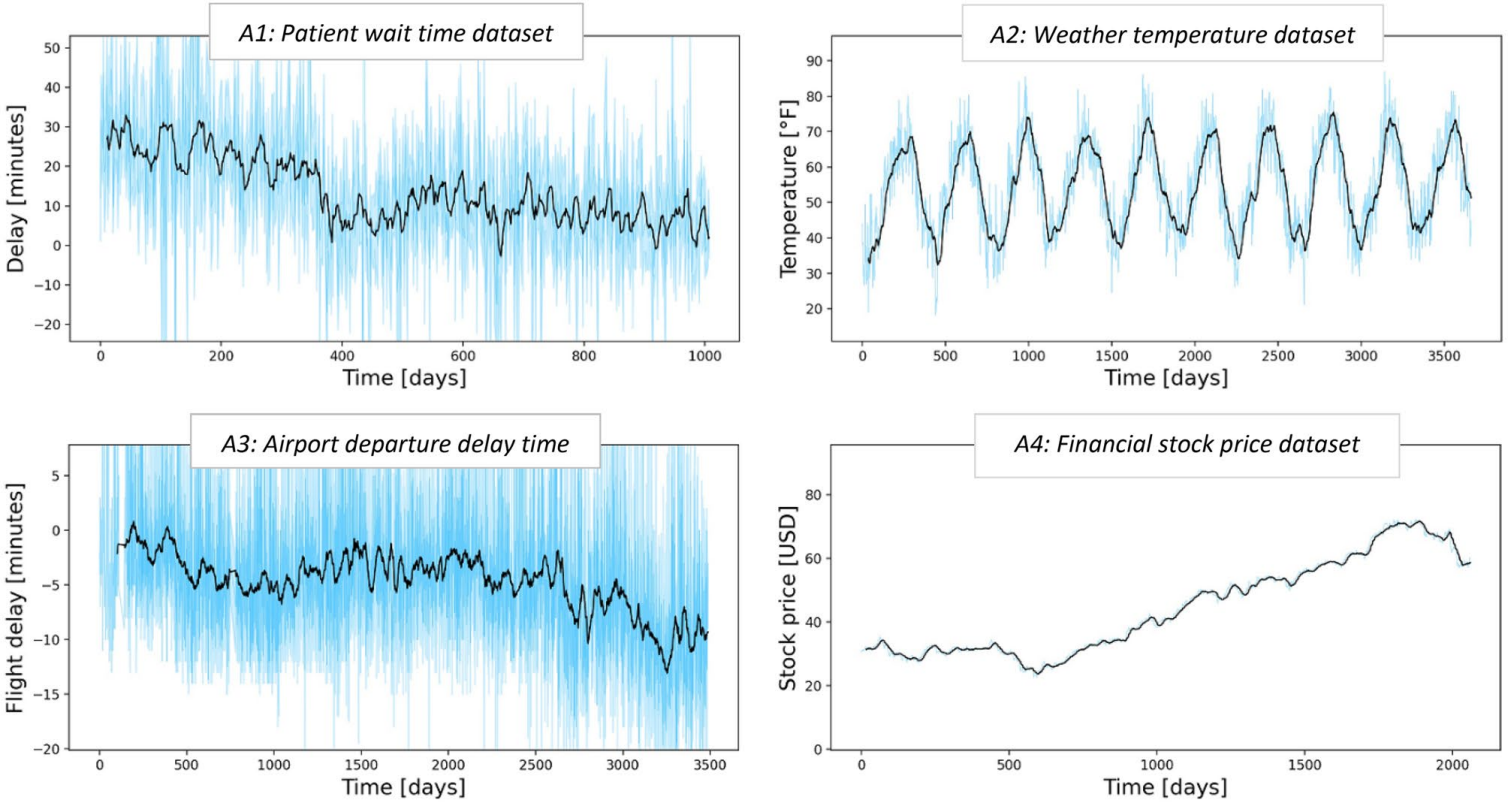
Examples of original data used in temporal degradation experiments. Each dataset timeline is shown on the horizontal axis (counting in days from the first dataset record) with its outcome metric on the vertical. When multiple datapoints were collected per day, they are shown with background color, and moving daily average curve. The data was taken from four different industries (healthcare, weather, airport traffic and financial), and the sets were chosen to contain no abrupt changes or gaps in their temporal data patterns.

To avoid any domain bias, we then chose 32 datasets from 4 industries, representing entirely different processes and target variables (Fig. 1):

1. *Weather* Predicting next day temperature and humidity (city of Basel, data range 2010–2020)^29^.
2. *Hospital operations* Predicting patient examination delay in 4 outpatient facilities in a busy regional hospital^30^.
3. *Airlines* Predicting flight departure delays in 15 domestic US airports^31^.
4. *Financial* Predicting next day stock closing value (11 stocks from S&P500).

We ensured that our analysis only involved datasets which (a) contained several years of timestamped data records, (b) did not contain missing/partial data, and (c) included several variables not directly derived from time (to consider the indirect impact of temporal patterns). We also ensured that each model-dataset pair produced a reasonable prediction quality, with cross-validated R^2^ in the 0.7–0.9 range at the time of model training. By doing so, we limited our work only to data/models with good initial quality—as it usually happens at model deployment time in practical settings.

Finally, we made sure that none of the above datasets contained any abrupt changes in the target variable’s value (Fig. 1). It is natural to expect significant reduction in the model quality when the underlying data changes abruptly. However, it is far more concerning to observe model quality degradation when the data remains consistent, with nothing alarming the users of potential problems. Thus, studying *model temporal sensitivity in the absence of major data drifts* presented one of the most interesting directions of our experiment.

All the procedures were performed in accordance with the relevant guidelines and regulations.

### Temporal degradation test

**Figure 2:**
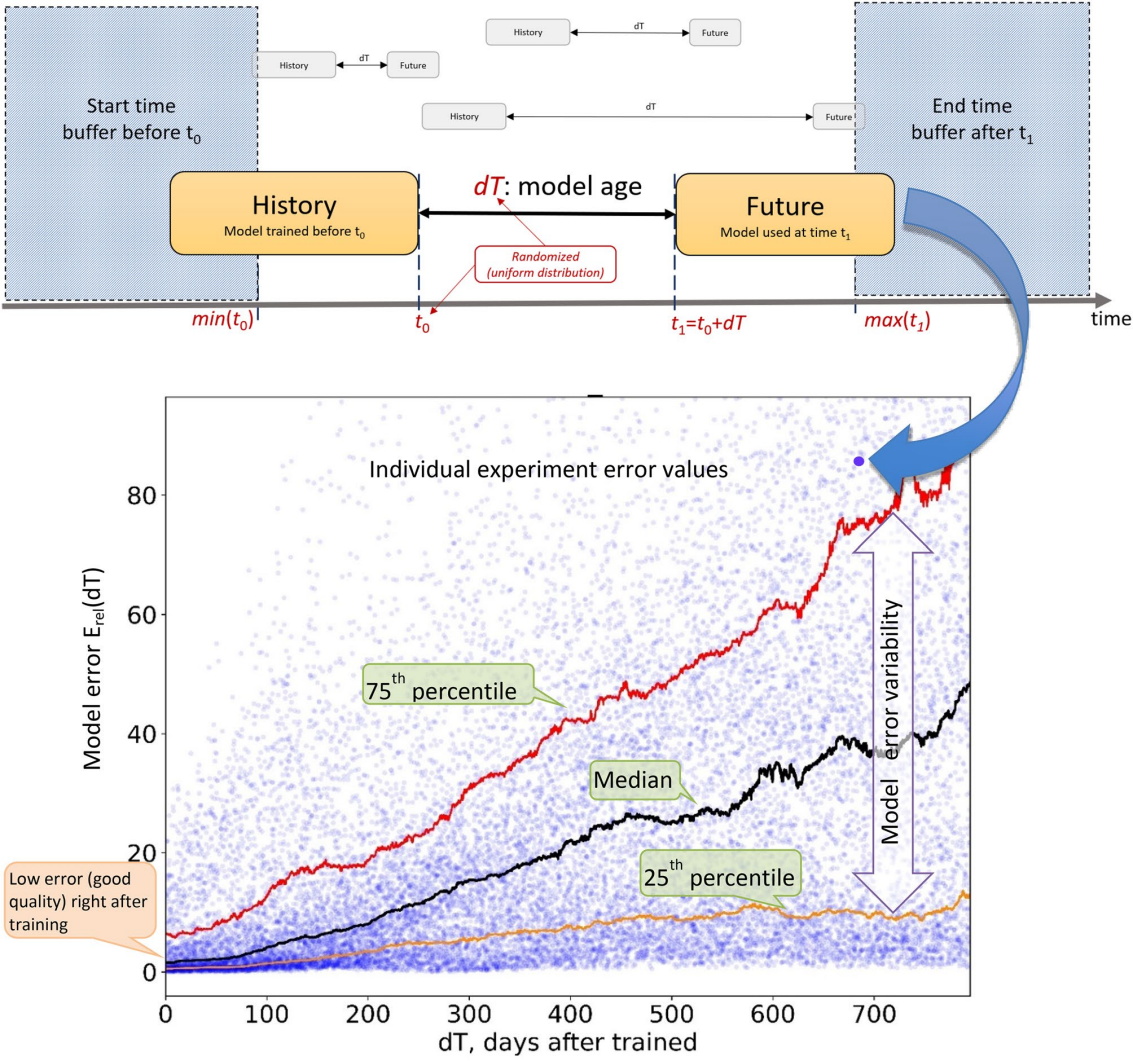
Top: AI temporal degradation experiment, modeling AI model deployment at a random date t_0_. The model is trained on the data (history) available at the deployment time t_0_. Each (Train,Test) pair is generated with uniform random distribution for dT and t_0_, and with Test dataset time starting dT days after the Train dataset ended. Two large time buffers are excluded on each end to ensure truly uniform sampling of dT and t_0_. Bottom: A typical layout of a model aging chart, with each small dot representing the (dT, E_rel_) outcome of a single temporal degradation experiment. One can observe the density of the original error distribution (small dots), as well as its principal trends (best-case with 25th percentile, median, and worst-case with 75th percentile). The gap between the best- and worst-case scenarios shows how the model quality variability changes with the model age dT.

We define the “age” of an AI model as the time passed since the model was last trained on then-current data. To identify the temporal degradation patterns in AI models, we designed an experiment imitating a typical AI model deployment—when the model is trained on the most recent available data right before the initial deployment time t_0_ (“history”), but used dT days later, at time t_1_ = t_0_ + dT (“future”). Using observation timestamps provided in each dataset, we ran a large number (N = 20,000) of individual “history-future” simulations, each corresponding to a single “model deployment instance” with randomly selected deployment time t_0_ and model age dT uniformly sampled from all possible (t_0_, dT) values (Fig. 2, top), to observe the change in the model quality with respect to the model age dT. To ensure that each model instance was given the same amount of training and testing data, we trained each model using one year of historical data (from the year ending at t_0_), and we excluded values from buffer time periods on each side of the data time range from the sampling distribution for t_0_ and dT.

To quantify this model performance change, we measured the standard mean squared error (MSE) at the time of model training as MSE(t_0_) = MSE_0_, and at the time of model evaluation as MSE(t_1_) = MSE(t_0_ + dT) = MSE_1_. Thus, MSE_0_ represented the model’s error on a test set produced at training time (“history”, initial model training), while MSE_1_ was the model’s performance on a new set of test data produced dT days after the model was trained (“future”, the time when model is used). We then computed and plotted the relative model error on these two test sets

$${E}_{rel}\left(dT\right)=\frac{MSE\left({t}_{1}\right)}{MSE\left({t}_{0}\right)}=\frac{MSE({t}_{0}+dT)}{MSE({t}_{0})},$$

as a function of model age dT, for each single deployment experiment. We intentionally started from zero or small negative dT, when model train and test sets touch or slightly overlap (Fig. 2, bottom)—expecting E_rel_(dT ≈ 0) to be close to 1.0, indicative of robust model performance at the time of training. Then we expanded dT to several months to see how E_rel_ values evolved with increasing model age dT > 0.

We have taken a few additional precautions to ensure more objective experimental outcomes:

- We have selected datasets with several years of consistently dense, continuous data, so that we can reliably measure model aging at any randomly-chosen time.
- We limited the maximum value of dT to one half of the entire dataset time range, to guarantee that uniform random sampling of t_0_ and dT can be maintained for all dT values, without aliasing the measurements or exceeding the time range.
- All models requiring random initialization were run with different random seeds, to exclude seeding bias.
- All modeling results, originally computed with Python, were verified with corresponding models provided in Matlab, to rule out any model implementation bias.

Finally, we ensured that all models were run with optimal or nearly-optimal hyperparameters, to exclude overfitting and to produce the most accurate predictions possible. Since retraining hyperparameters for each individual model run was not feasible due to the large number of numerical experiments N, we compromised by recomputing the optimal hyperparameter values after each 100 experiments, on more specific subranges of t_0_ times. In addition to this, each hyperparameter training was done by using extensive cross-validated grid search with fivefold, and time series train/test data splitting (more appropriate for temporal predictions)—thus resulting in more “time resistant” hyperparameter optimization. As a result, we were able to find the optimal regularization penalty coefficients for the RV, XG and NN models, the optimal number of trees and tree depths for the XG and RF models, and the optimal layout (number and sizes of hidden layers) and activation functions for the NN models. The sizes of both the train and test sets were fixed to provide comparable error metrics.

Using this approach for each choice of dataset and each machine learning model applied to it, we computed the “model aging” chart, as illustrated in Fig. 2 (bottom). There, each point represents a single experiment result (model error E_rel_(dT), plotted against its dT value), and the three curves show moving error percentiles (25th, 50th (median) and 75th). Thus, the original points provide the most granular view of the model error density distribution, while the moving percentile curves reflect the best-case (25%), median (50%), and worst-case (75%) trends in this error, as functions of the model age dT.

## Results

We performed our experiments for all 4 × 32 = 128 (model, dataset) pairs, and we observed temporal model degradation in 91% of cases. In this section, we classify our results into several principal degradation patterns of varying complexity and discuss their impact on AI model implementation.

### Major degradation patterns: from moderate to explosive

**Figure 3:**
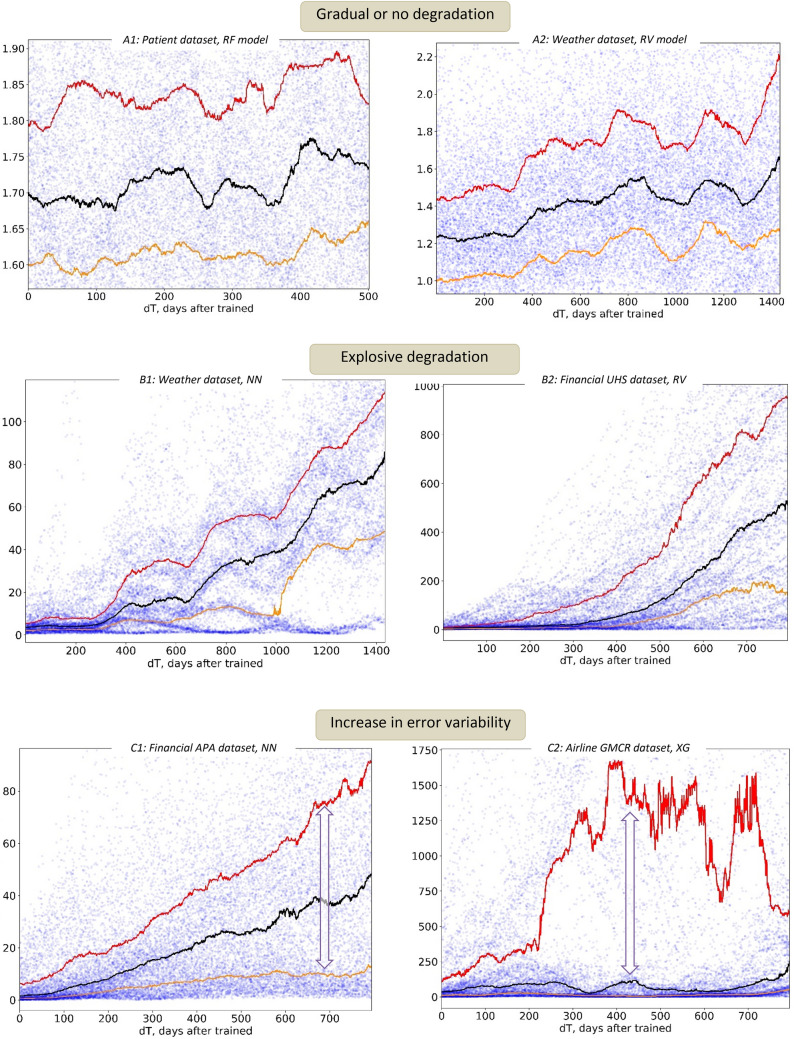
(**A**) Gradual AI model degradation patterns, with relative model error E_rel_ increasing no faster than linearly over time. Note that occasional reversal of aging (decrease in E_rel_ values) is possible as well. (**B**) Explosive AI model aging patterns (in data with very moderate and predictable growth). (**C**) Increasing unpredictability AI model aging patterns (in data with very moderate and predictable growth).

Figure 3 provides the baseline classification of the major observed temporal degradation patterns. The relative MSE error E_rel_(dT) is shown on the vertical axis, as a function of the model age dT (horizontal axis). Note that for all cases, the E_rel_ median for small dT values is close to 1.0, which means that the model was providing high predictive quality right after its training (deployment).

The results in Fig. 3 lead to a few important observations.

First, temporal degradation in AI model quality presents a serious challenge which cannot be explained by the temporal drifts in the underlying data alone: some models can capture drifting processes very well, thus showing no significant signs of temporal degradation. This can be seen in the A1 and A2 examples (Fig. 3), where the patient traffic and weather model errors remain virtually unchanged for several years—despite serious outliers and downward drift in the patient data, and significant seasonal shifts in the weather data (Fig. 1). These and similar results demonstrate that *data drifts alone cannot be used to explain model failures, or to trigger model quality checks and re-training*. Instead, temporal degradation of AI models represents a completely different phenomenon, not solely driven by the drifts in the data, and not necessarily predictable based on these drifts.

Second, temporal model degradation can develop gradually, but can also escalate very abruptly, after a significantly long period of good model performance (Fig. 3B1,B2,C2). Moreover, this “breakage point” cannot be explained by any particular change in the data: for example, the NN model (Fig. 3B1) began to degrade after performing accurately for a year, while the RV model (Fig. 3A2) remained consistently accurate—on the same dataset, with the same choices of train and test data. Thus, *temporal model quality depends on the choice of the ML model, and the model stability on a certain set of data*.

Finally, temporal increase in the model error may not be the only sign of its degradation. Some models can perform reasonably well “on average”, but the variability of their error values can significantly grow or fluctuate with time. This can be seen in Fig. 3C, where the gap between the 25th and 75th model percentiles (best- and worst-case model performance) significantly changes with time. Error variability degradation can be even more challenging in practical AI implementations: the reasonably low median model error may still create the illusion of an accurate model performance, while the real model outcomes would become less and less certain.

These examples highlight the challenges that complex temporal AI patterns pose for implementing practical AI quality controls. As we can see, neither the data nor the model alone can be used to guarantee a consistent predictive quality or long-term accuracy. Instead, the temporal model quality is determined by the stability of a specific model, applied to the specific data, at specific time. At this point, very little is known of the numerical stability properties of ML models, and virtually no effort has been made to advance this critically important area. As a result, the missing awareness of model failure patterns and the lack of robust stability controls make most models too fragile to run for a long time without retraining, failing with extremely inaccurate results on even the most consistent data.

### Complex degradation patterns

While the previous section presented the most common model degradation patterns identified by our experiment, we have also observed several instances of more complex degradation behavior, presented below. These patterns would be much harder to detect with simple error thresholds, therefore presenting more alarming challenges to practical AI deployments.

#### Strange attractors and chaos

**Figure 4:**
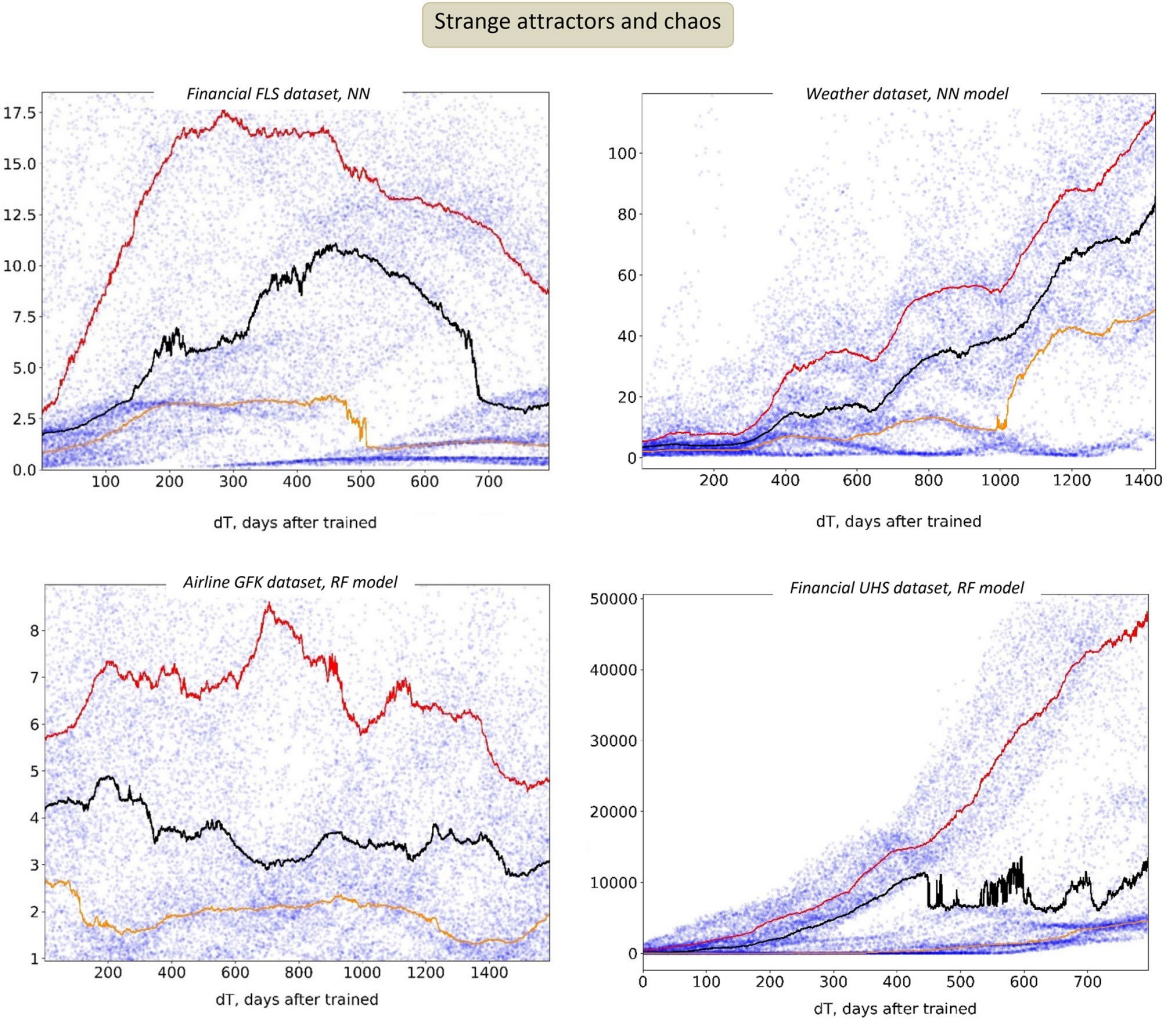
Strange attractors, as dense areas of most probable errors, in temporal model degradation.

*Strange attractors* can be described as the sets of more probable states toward which a complex system tends to evolve^32^. Extensively studied in chaos theory, these attractors are often found in dynamic systems, and we observed attractor-like behavior in our study, when the model error distribution developed very non-uniform, dense basins of highly probable errors. This nontrivial behavior can be seen in Fig. 4, where the individual dots, representing model E_rel_ errors from different randomized experiments, begin to form clusters of most probable values.

**Figure 5:**
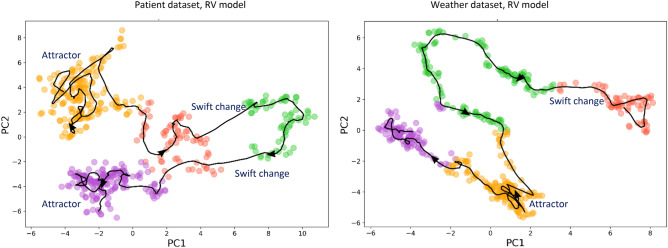
“Phase portraits” of evolving AI models. Model temporal evolution, visualized as a curve in the model feature importance space (two major principal components, PC1 and PC2). Each marker point represents the feature importance values of the model when trained at a specific time, and the curve shows the overall temporal path of the model. One can clearly see temporal clustering of the model feature values (shown with different colors). Note how the model can be attracted to certain temporal clusters, where it spends significant time, and then can swiftly change to another state.

This degradation pattern corresponds to one of the most practically concerning scenarios, when AI model outputs and errors, evolving over time, can get trapped in several most probable states, erratically switching between them. It also highlights the complex dynamic-system-like nature of temporal AI models, suggesting the application of dynamic system analysis. In particular, we would like to recommend the use of *phase portraits*^33^ to visualize and study the temporal trajectories of AI models in the space of their feature importance values (computed at different time points). Figure 5 provides an example of our approach, showing the temporal path of an ML model, visualized in its own feature space (projected onto the first two major principal components). In this “phase portrait”, one can clearly observe very visible clustering of the model feature importance values, and the model temporal evolution as a path through these clusters. Note how the model tends to be “attracted” to different clusters for substantial periods of time, but then transition to another attractor cluster.

Undoubtedly, these elements of dynamic chaos would be much harder to detect and to measure, leading to serious misinterpretation of model results and quality^34^; temporal AI models should become a subject of serious mathematical research.

#### Evolving bias

In recent years, significant efforts have been dedicated to making machine learning models fairer and more unbiased. However, the models, initially checked to ensure fairness, can significantly diverge from this optimal state as they age.

**Figure 6:**
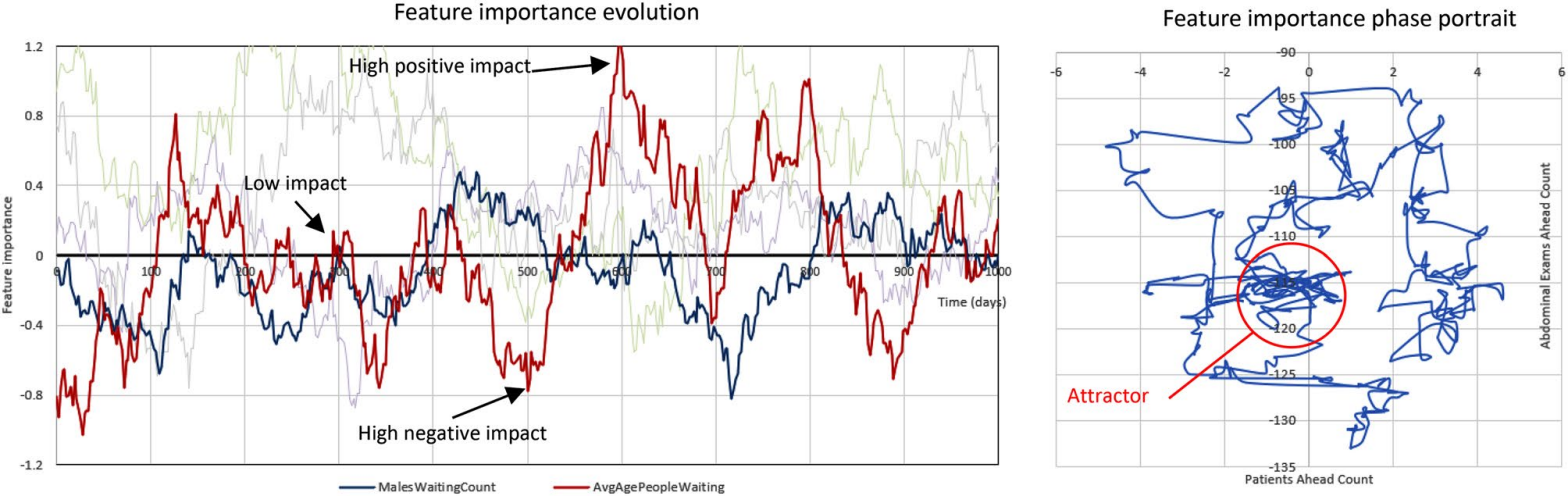
Left: Feature contributions to the final model outcome, changing in time (ridge regression model, patient wait time dataset). We used a 3-month sliding window along the time axis, computing normalized feature importance values for each window position. As a result, the plot visualizes the temporal evolution of individual feature impacts on the model outcome, with two features shown in the foreground, and more in the faded background. Right: Model temporal path, traced in the phase space of its two feature (importance) values. Note another attractor area in the center, and the overall circular shape of the model path, significantly changing between the two features.

This bias-evolving behavior can already be recognized in Fig. 5, where the model travels between sometimes very densely clustered sets of its feature values. This means that different feature value clusters can dominate the model outcome at different times, thus leading to disproportional, biased feature impact on the model’s predictions. Figure 6 provides another example of how a few principal features of the patient wait time model can change their importance depending on the dates when the model was trained. Although the features were normalized for comparison, one can clearly observe the significant temporal changes in their contributions to the predicted model outcome. Moreover, these contributions can vary not only in their magnitude, but in their sign as well. As a result, in the Fig. 6 (left) example, both the average age of the waiting patient and the patient’s gender can impact the model outcome negatively, positively, or not at all, depending on the time when the model was trained. Figure 6 (right) demonstrates the same phenomenon in the model phase portrait, presented in the space of two feature importance values: complex, sometimes chaotic, and overall very evolving feature impacts on the final model outcome value.

This leads to another important observation. Very often, feature importance values are computed to aid model reduction, or to identify the principal drivers of a process^35–37^. However, as our results demonstrate, this should be done with extreme caution, and taking the temporal component into account. The selection of the most important features will largely depend on the timeframe of the data used for this selection, and on the age of the model. As a result, model feature contributions—and therefore model fairness—are not static and can significantly evolve over time.

#### Latent features and seasonality

Many real-life processes can be impacted by powerful yet hidden variables, significantly contributing to the temporal model evolution. Biological clocks, activity cycles, seasons and even regular equipment upgrades can easily alter the quality of AI models and result in significant model degradation.

**Figure 7:**
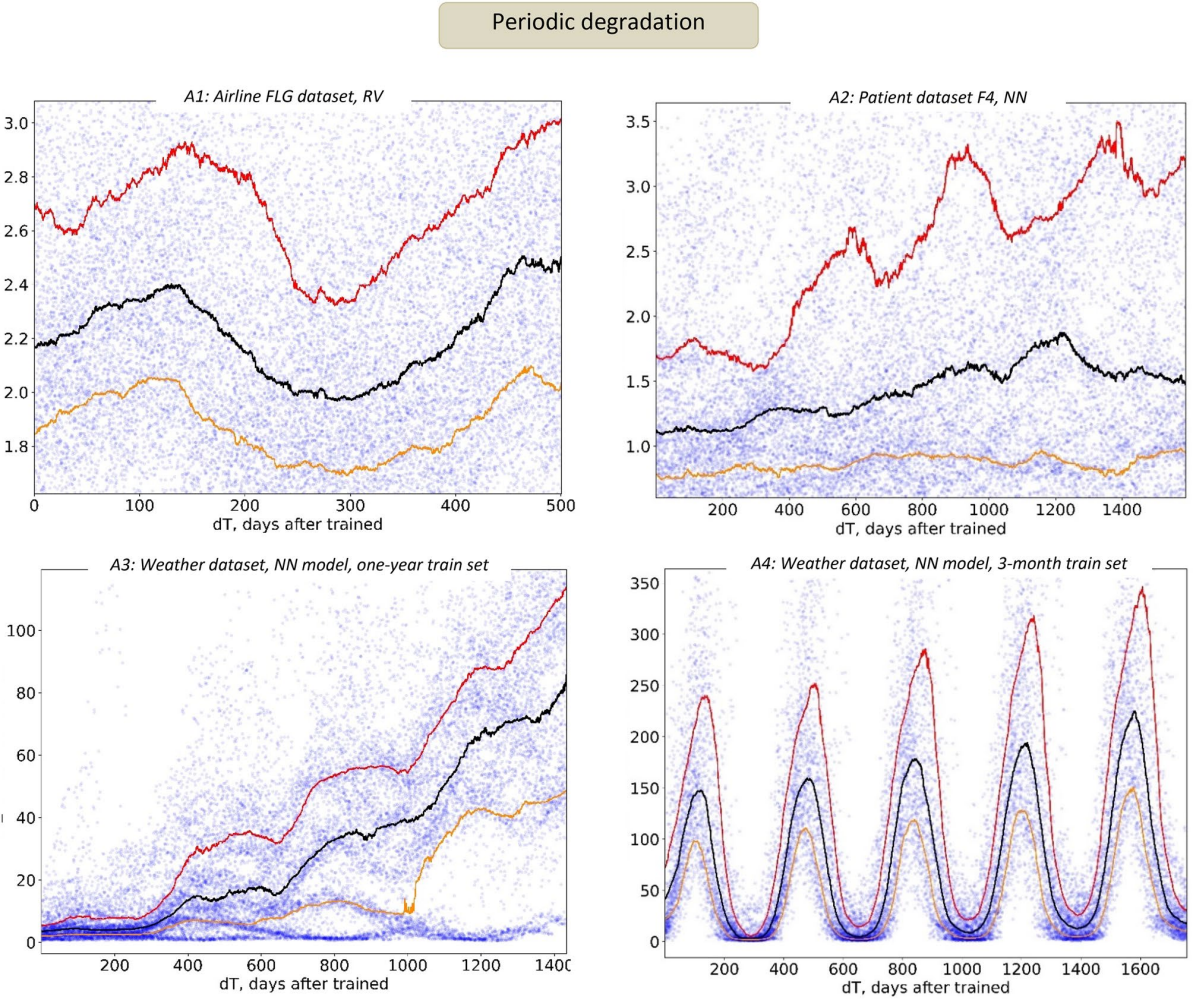
Periodic temporal degradation in AI models. Note how using different sizes of the train set affects periodicity in the model error (bottom row).

One of the most omnipresent of these factors is seasonality, and oscillating error patterns can be seen in some of our examples reported above. Figure 7 provides a few more noteworthy examples of seasonal changes in the AI model quality. Cases A1 and A2 are particularly interesting, because their original data did not exhibit any signs of seasonality (airport and patient datasets, Fig. 1), and their prediction targets (next flight delay, or next patient wait for an examination) were extremely short-term, typically well under an hour. Yet one can clearly see the annual, 365-day periodicity in both the A1 and A2 plots in Fig. 7.

The bottom row in Fig. 7 provides another important example of how latent seasonal patterns can impact temporal model quality. In this case, the same neural network model was trained on the same weather dataset using a full year (A3), and 3 months (A4) of training data. As one can observe, shortening the training history timeline locked the model into a much more periodic error pattern, which also produced higher peaks in E_rel_ values compared to the model trained on the full year.

One seemingly obvious remedy would be increasing the train set size as much as possible, to capture the most possible latent process contributors. However, this will run into the risk of including more outdated data, producing a less adequate model. As a result, achieving the right balance between “recent enough” and “large enough” training data represents a serious challenge in the dynamic nature of temporal AI. On the other hand, in many AI applications the size of the history dataset is often determined by what is practically available (and not by what is optimal), and missing the right seasonal cycle can significantly impact the model quality.

## Discussion

### Causes of temporal degradation

As our results demonstrate, temporal degradation of AI model quality can manifest itself in many different hidden and non-trivial ways. Although the complete study of this complex phenomenon lies beyond the scope of this paper, we would like to make a few principal observations based on our results.

First, although temporal degradation is often triggered by progressive data drifts, these two concepts are completely different. Unlike concept drifts, which reflect temporal data changes on the absolute timeline, AI aging describes how various AI models change *relative to the time of their training*, whenever the training might have happened. In our experiment (Fig. 2, top), we considered models trained at different random timepoints, thus minimizing the impact of the global data drifts, yet many models still developed strong degradation patterns.

Second, temporal degradation can be driven by many additional factors, such as the choice of model hyperparameters or training set size (Fig. 7, bottom). That is, even when using exactly the same model design with the same data, one can produce models with completely different temporal degradation behavior due to selected settings, properties, and even random seeds.

This observation can be extended even further, if one considers different models applied to the same dataset. As was already illustrated in Fig. 2 (cases A2 and B1), two models, trained on the same dataset with the same history size, can demonstrate completely different temporal degradation patterns, from barely visible (A2) to explosive (B1). Thus, one should not presume that all temporal drifts in the data will inevitably break their models. Instead, the choice of the model and its stability becomes one of the most critical factors in dealing with temporal degradation.

All of this makes us believe that the true causes of AI aging should be corrected in the models themselves, and not in the data. Just like many other numerical algorithms, machine learning models can be unstable and sensitive to change—which, when applied to certain data values, can produce extremely inaccurate results. The further study of this phenomenon lies well beyond the scope of this paper, but we would like to make the first step by classifying its patterns and their magnitude.

### Potential solutions to temporal degradation problem

Retraining a model on a regular basis looks like the most obvious remedy to AI aging, but this is only simple in theory. To make retraining practically feasible, one needs to, at least:

- develop a trigger to signal when the model must be retrained;
- develop an efficient and robust mechanism for automatic model retraining, and
- have constant access to the most recent ground truth.

These steps require serious changes in our current AI deployment practices.

**Figure 8:**
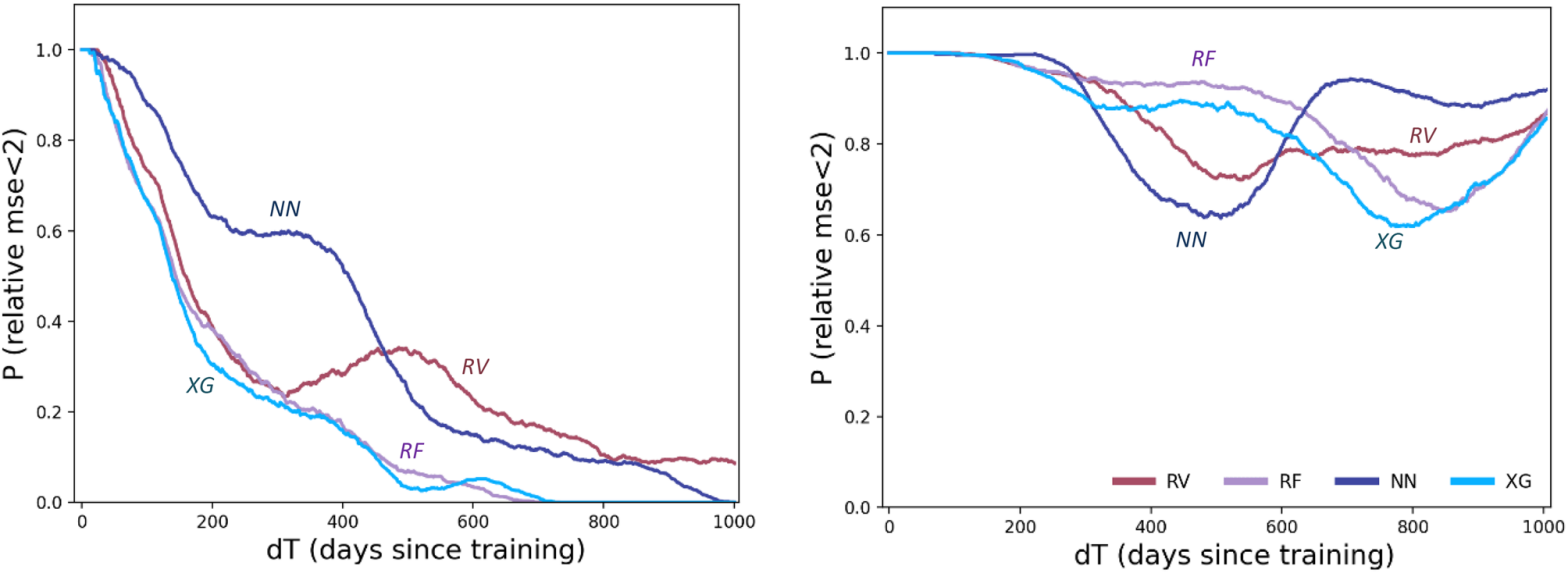
Probability P_ac_(dT) of different models performing accurately as a function of time (maintaining E_rel_ < 2.0), on two sample datasets. Note the variability in degradation patterns between different models, and different datasets.

First, the retraining trigger can be implemented only when we gain enough knowledge of possible AI aging patterns, to detect them consistently and as early as possible; however, triggering on some of the error patterns reported in our study presents a non-trivial task. Some of these multifactorial triggering challenges can be visualized and studied by exploring the probability P_ac_(dT) of retaining an accurate model, which we can define as maintaining E_rel_ < 2.0 (keeping MSE below double its value from training time). Figure 8 shows P_ac_(dT) function for four major ML model types, on two sample datasets from our experiments.

As one can see, P_ac_ is not guaranteed to be high even for relatively small dT, and depends on many circumstances (such as specific time of training t_0_ or model hyperparameters). As model age dT begins to increase, the inevitable temporal decline in P_ac_(dT) becomes even more pronounced, aggravated by specific model selection, data periodicity, and more. Triggering a specific model degradation event will require controlling for all of these factors.

Second, model retraining, which clearly needs to be automated to be done frequently and on demand, may run into many practical challenges such as “catastrophic forgetting”^34^, lack of convergence, suboptimal changes to the training parameters, or even model dependency on the random seed^38^. This is why many high-risk industries—such as healthcare—have preferred to avoid automated model retrains^26,39^. As a result, AI practitioners are facing the choice between two evils: skipping model retraining to sidestep its risky side-effects, or running it with serious liability for anything that may go wrong. This is further complicated by the fact that current model retraining, as we know it, can be a very time-consuming and laborious task, relying on additional sources of ground truth, manual data labeling, and large data volumes^22^. Reproducing this approach in an automated, self-learning manner will take a considerable effort, and in some cases may not be possible at all.

## Conclusions

Our study contributes an introduction and exploration of the phenomenon of temporal ML model degradation—a virtually unknown, yet critically important, property of machine learning models, essential for our understanding of AI and its applications. Using extensive numerical simulations, designed to reproduce a typical deployment cycle of ML models, with varying model and data types, we demonstrate the difference between ML degradation and data concept drifts, reveal major degradation patterns and risks, and discuss practical challenges and potential solutions. We show that AI models do not remain static, even if they achieve high accuracy when initially deployed, and even when their data comes from seemingly stable processes.

Therefore, significant efforts should be dedicated to understanding and reducing temporal degradation. To begin, we would recommend the following:

1. Viewing, studying, and testing temporal AI models as *dynamic systems,* with significant attention given to their stability, “phase portrait” patterns, and temporal errors (Figs. 5, 6).
2. Performing temporal degradation tests, where not only model quality, but also model temporal stability and error distribution patterns are verified to assure reliable temporal performance (Figs. 2, 8).
3. Using temporal degradation tests to select not only the most stable model for a given task, but also the best choice of model hyperparameters and train size (Fig. 7).
4. Evaluating model feature importance values over time and excluding the features with the most erratic behavior to increase stability (Fig. 6).
5. When the model is implemented in production, measuring model error values as frequently as possible, and automatically alerting model users when these errors exceed acceptable thresholds (Fig. 8).

We are also certain that more in-depth research needs to be done to understand how to make temporal models more stable. This can be achieved by applying concepts already developed in other areas of computational mathematics, such as the use of stability (Lyapunov) exponents in differential equations and numerical methods^40^. This will be particularly important with “black box” models (such as deep-learning neural networks), where the failure to converge to a time-stable state can be exacerbated by high-dimensional parameter spaces, as has been already shown in computer vision^12^ and natural language processing^13^. In essence, *any machine learning model or feature should be viewed as a function of time*. And as a function of time, it can produce completely different results depending on when it was trained and when it was used.
